# Long-term analysis of hematological parameters as predictors of recurrence patterns and treatment outcomes in cervical cancer patients undergoing definitive chemoradiotherapy

**DOI:** 10.1007/s00066-024-02278-8

**Published:** 2024-08-05

**Authors:** Aysenur Elmali, Ozan Cem Guler, Birhan Demirhan, Melek Yavuz, Cem Onal

**Affiliations:** 1 Faculty of Medicine, Department of Radiation Oncology, Baskent University, Ankara, Turkey; 2 Faculty of Medicine, Adana Dr. Turgut Noyan Research and Treatment Center, Department of Radiation Oncology, Baskent University, 01120 Adana, Turkey; 3 Division of Radiation Oncology, Iskenderun Gelisim Hospital, Hatay, Turkey

**Keywords:** Cervical cancer, Radiotherapy, Neutrophil-to-lymphocyte ratio, Platelet-to-lymphocyte ratio, Hemoglobin

## Abstract

**Purpose:**

This study sought to determine the predictive and prognostic value of clinicopathological parameters and neutrophil-to-lymphocyte ratio (NLR), platelet-to-lymphocyte ratio (PLR), and hemoglobin (Hgb) level in predicting recurrence patterns and locoregional relapse-free survival (LRFS) and distant metastasis-free survival (DMFS) in cervical cancer patients receiving definitive chemoradiotherapy (ChRT).

**Methods:**

This study included 261 cervical cancer patients treated with ChRT. The primary endpoints were the predictors of local recurrence (LR) and distant metastasis (DM), whereas the secondary endpoints were LRFS and DMFS. The association of survival with potential prognostic factors was analyzed using Cox regression analysis, and the predictors of LR and DM were identified using logistic regression analysis.

**Results:**

The median follow-up time was 10.9 years. Recurrences occurred in 132 patients (50.6%) within a median of 11.2 months after definitive ChRT. NLR and PLR values were significantly higher in patients with LR and DM than in those without, with no significant differences in Hgb levels in patients with or without LR and DM. In the multivariable logistic regression analysis, lymph node metastasis, elevated NLR, and low Hgb level were significantly correlated with LR and DM. In the multivariable analysis, large tumor size, presence of lymph node metastasis, and elevated NLR were the independent predictors for poor LRFS and DMFS, and Hgb level was an additional prognostic factor for DMFS.

**Conclusion:**

Hematological markers, particularly NLR and Hgb, may serve as cost-effective and readily accessible indicators for predicting recurrence and survival in cervical cancer patients, contributing to their practical use in routine assessments.

## Introduction

Cervical cancer remains to be a major global health problem and is the fourth most common cancer among women worldwide [1]. The mortality rates for cervical cancer are high, and predicting its prognosis may pave the way for more individualized treatment approaches. While clinicopathological factors such as tumor size, lymph node status, histological type, tumor grade, and lymphovascular space invasion have been validated as prognostic and predictive factors in cervical cancer, identifying and validating novel factors could further improve prognosis and treatment outcomes [2, 3].

In an effort to identify additional prognostic indicators, hematologic parameters have been investigated given the dynamic interaction between tumor-induced inflammation and host immune response. Cancer patients with systemic inflammation typically show inferior survival outcomes compared with those without inflammation [4, 5]. Studies have indicated that certain hematologic parameters, including neutrophil-to-lymphocyte ratio (NLR), platelet-to-lymphocyte ratio (PLR), and elevated C‑reactive protein level, are potential indicators of systemic inflammation associated with cancer [6–8]. A meta-analysis encompassing 14 studies and involving more than 6000 patients has confirmed the independent prognostic significance of pretreatment NLR on survival, specifically in cervical cancer [9]. Moreover, studies investigating the relationship between PLR and cervical cancer prognosis have suggested that an elevated PLR is associated with poor treatment outcomes [10, 11]. Anemia is an adverse prognostic factor for cervical cancer, and it impairs oxygen transport due to low hemoglobin (Hgb) levels, potentially jeopardizing the efficacy of radiation therapy (RT). Moreover, it has been suggested that anemia could serve as an indicator of cancer progression and disease-related complications [12].

The prognostic implications of hematologic parameters in cervical cancer have been extensively studied, and certain characteristics of hematological tests render them particularly useful in clinical practice. However, the majority of the existing studies have investigated the impact of hematological parameters on survival, with a diverse patient population and a short follow-up period. Moreover, information is limited regarding the relationship between hematological parameters and recurrence patterns in cervical cancer patients who received definitive chemoradiotherapy (ChRT). This study therefore aimed to investigate the impact of prognostic factors on predicting local recurrence (LR) and distant metastasis (DM) in cervical cancer patients who underwent definitive ChRT and were followed up for a long period of time. Moreover, this study examined the prognostic factors associated with local recurrence-free survival (LRFS) and distant metastasis-free survival (DMFS).

## Methods

### Patient selection

The clinical data of 261 cervical cancer patients treated with definitive ChRT between October 2006 and September 2014 were retrospectively analyzed. The inclusion criteria were as follows: (i) patients with a confirmed histological diagnosis of squamous cell carcinoma (SCC), (ii) those followed up for a minimum of 5 years, and (iii) those who received external beam radiotherapy (EBRT) and intracavitary brachytherapy (BRT). The exclusion criteria were as follows: (i) patients who had undergone surgery, (ii) those with distant metastases at the time of diagnosis, and (iii) those with hematological disease or who have undergone blood transfusion prior to hematological parameter evaluation or other conditions that cause changes in hematological parameters.

All patients were staged using the 2018 International Federation of Gynecology and Obstetrics (FIGO) system. All hematological tests were performed prior to treatment with ChRT. The pretreatment NLR and PLR values were calculated as the ratio of neutrophil and platelet counts to the lymphocyte count, respectively. This calculation relied on the absolute counts for neutrophils, lymphocytes, and platelets.

This study was approved by the Institutional Review Board of Baskent University (Project No. KA13/146). The requirement for a written informed consent for all patients was waived due to the retrospective nature of this study.

### Treatment protocol

The patients were treated with three-dimensional conformal EBRT (140 patients, 53.6%) or intensity-modulated RT (121 patients, 46.4%) combined with weekly cisplatin (40 mg/m^2^) followed by intracavitary high-dose-rate BRT, as previously described [13]. The primary cervical tumor, the entire uterus, and the regional pelvic lymphatics were all irradiated. In patients with para-aortic lymph node metastasis, a para-aortic field was added. A total dose of 50.4-Gy of external radiation therapy (using 6‑MV photons) was administered daily, with a dose of 1.8 Gy per treatment fraction, from Monday to Friday. Following that, intracavitary brachytherapy was administered in four fractions, with a dose of 7 Gy per fraction, given twice weekly, targeting a specified minimum area. During each high-dose rate brachytherapy session, a CT scan of the pelvis was performed using a 2.5-mm slice thickness. The CT scan was done with a CT compatible applicator in place. The delineation of the target volume was determined using CT data obtained during the time of BRT, with additional support from clinical and radiographic findings.

### Follow-up

The patients were monitored on a regular basis, with follow-ups scheduled every three months for the first two years, then every six months until year five, and finally annually. In addition, detailed physical and gynecologic examinations were performed, along with routine complete blood cell counts, serum biochemical testing, and imaging studies. For the assessment of treatment response, magnetic resonance imaging (MRI) or fluoro-deoxyglucose positron emission tomography (FDG-PET/CT) scans were performed at least three months after completion of definitive ChRT. Biopsies were not conducted as a routine procedure, except in cases where suspicious lesions were identified.

### Statistical analysis

SPSS 26.0 (SPSS for Windows; IBM Corp.) and GraphPad Prism version 10.1.2 (San Diego, California, USA) were used for statistical analysis. Differences in patient and tumor characteristics were analyzed using χ^2^ test or Student’s t‑test. The primary endpoints are the predictive factors for LR and DM. The secondary endpoints were LRFS, which refers to the duration between diagnosis and local or regional recurrence, death, or the latest follow-up, and DMFS, which refers to the duration between diagnosis and distant metastasis, death, or the latest follow-up. Univariate logistic regression was applied to identify the predictors of LR and DM, and the logistic regression analysis included the possible factors identified in the univariate analysis for the multivariate analysis. The LRFS and DMFS rates were calculated using the Kaplan-Meier method. Receiver operating characteristic (ROC) analyses were conducted to establish the optimal cut-off points for hematological parameters, enhancing the accuracy of the predictive models. In the univariate analysis, the log rank test was used. Covariates with *p* < 0.05 in the univariate analysis were included in the Cox proportional hazards model for the multivariate analyses. A *p *value of > 0.05 indicated statistical significance.

## Results

### Patient characteristics

Table 1 summarizes the patient and tumor characteristics of the subjects (*n* = 261). The median age of the entire cohort was 57 years (range, 19–89 years). The majority of the patients (86.2%) had locally advanced disease (stage ≥IIB) with nearly half of them (51.3%) having regional nodal metastasis. The majority of the patients (81.6%) were staged with FDG-PET/CT, whereas nearly half (50.2%) underwent MRI scans for staging purposes.

**Table 1:** Patient, tumor, and treatment characteristics

Characteristics	Number	%
*Age, years (median, range)*	57 (19–89)	
*Tumor size, cm (median, range)*	5 (2–13)	
*FIGO stage*		
IB2	23	8.8
IIA	13	5.0
IIB	129	49.4
IIIA	19	7.3
IIIB	68	26.1
IVA	9	3.6
*Lymph node metastasis*		
No	127	48.7
Yes	134	51.3
*Pelvic MRI*		
Present	131	50.2
Absent	130	49.8
*FDG-PET/CT*		
Present	213	81.6
Absent	48	18.4

Abbreviations: *FIGO* International Federation of Gynecology and Obstetrics, *MRI* magnetic resonance imaging, *FDG-PET/CT* fluorodeoxyglucose positron emission tomography

The median total EBRT dose was 50.4 Gy (range, 45–54 Gy) and the total intracavitary BRT dose was 28 Gy delivered in four applications. All patients received ChRT, with median concurrent chemotherapy (ChT) cycle of 6 (range, 2–6 cycles), and 212 (81.2%) patients received at least four cycles of concurrent ChT.

### Hematological parameters

The median Hgb level and the absolute neutrophil and platelet counts were 11.4 g/dL (range, 7.0–15.4 g/dL), 7.3 cells/µL (range, 1.6–28.4 cells/µL), and 273 × 10^9^/L (range, 110–600 × 10^9^/L), respectively. The median NLR and PLR values were 2.9 (range, 1.0–13.9) and 13.0 (range, 3.6–73.7), respectively.

**Fig. 1:**
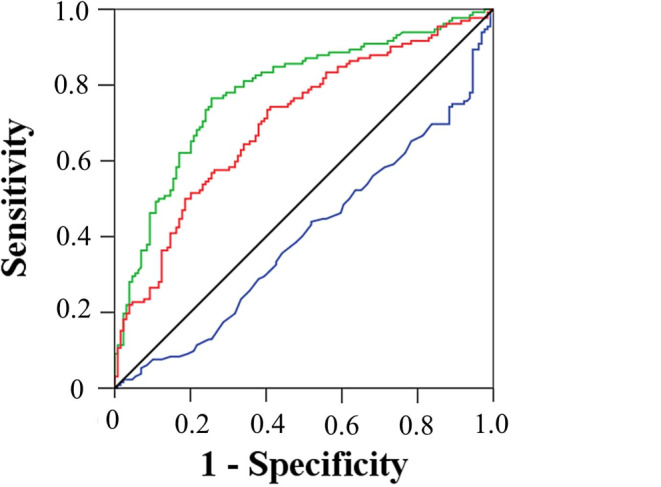
Receiver operating characteristic curve analysis of recurrence prediction based on neutrophil-to-lymphocyte ratio (green line), platelet-to-lymphocyte ratio (red line), and serum hemoglobin (Hgb) level (blue line). (Black line represents reference line)

Figure 1 shows the ROC curve analysis of the NLR, PLR, and Hgb level for the prediction of disease progression. The area under curve (AUC) for NLR was 0.784 (95% CI, 0.729–0.833; *p* < 0.001), and 2.92 was the cutoff value for NLR with a sensitivity of 76.5% and a specificity of 74.4%. For PLR, the AUC was 0.702 (95% CI, 0.642–0.756; *p* < 0.001), and the cut-off value for predicting the disease progression at 73.5% sensitivity and 59.7% specificity was 11.35. The AUC for Hgb was 0.601 (95% CI, 0.539–0.661; *p* = 0.004), with a cut-off value of 9.82 g/dL (sensitivity, 30.3%; specificity, 88.4%).

### Treatment outcomes

**Fig. 2:**
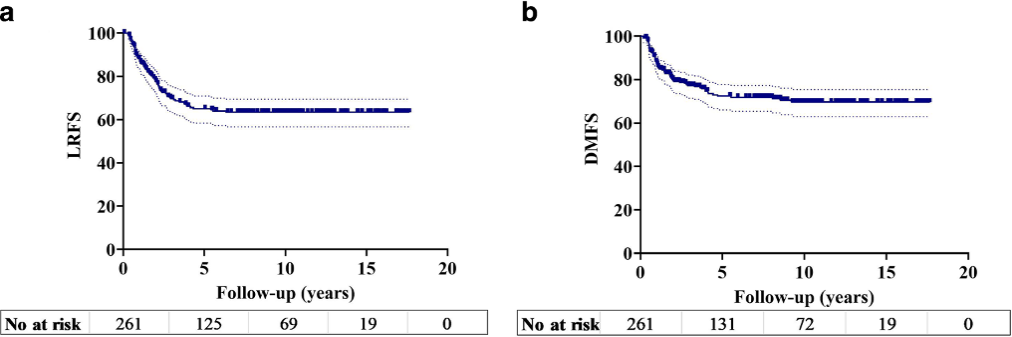
Kaplan-Meier graph demonstrating (**a**) locoregional relapse free survival (LRFS) and (**b**) distant metastasis free survival (DMFS)

The median follow-up was 10.9 years (interquartile range (IQR), 9.8–11.9 years). The 5‑year LRFS and DMFS rates were 72.4 and 69.6%, respectively, and the corresponding 10-year rates were 65.1 and 63.4% (Fig. 2). At the time of the most recent follow-up, 97 patients (37.2%) were still alive (4 patients [1.5%] with disease), whereas 150 patients (62.3%) had died (128 patients [49.0%] due to disease progression).

Disease progression was observed in 132 patients (50.6%) within a median of 11.2 months (range, 1.0–107.5 months) after ChRT completion. Of these patients, 48 (18.4% of the total) had DM only, 44 (16.9%) had LR only, and 20 (7.7%) had both DM and LR. Of these recurrences, 53.0 and 75.8% occurred within one and two years after completion of ChRT, respectively.

### Correlation between parameters

The NLR (4.84 ± 2.42 vs. 3.28 ± 1.95; *p* < 0.001) and PLR (10.82 ± 3.27 vs. 14.28 ± 8.27; *p* = 0.001) values were significantly higher in patients with LR than in those without LR. Similarly, NLR (4.83 ± 2.72 vs. 3.41 ± 1.90; *p* < 0.001) and PLR (19.32 ± 11.01 vs. 14.89 vs. 9.60; *p* = 0.007) values significantly differed in patients with and without DM. However, no significant differences in Hgb levels were observed between patients with and without LR (11.1 ± 1.7 g/dL vs. 11.5 ± 1.6 g/dL; *p* = 0.11) and between patients with and without DM (11.1 ± 1.7 g/dL vs. 11.4 ± 1.4 g/dL; *p* = 0.12).

According to the univariate logistic regression analysis, age, tumor size, FIGO stage, lymph node metastasis, NLR, PLR, and Hgb level were significantly correlated with LR and DM (Table 2). In the multivariate regression analysis, regional lymph node metastasis and changes in NLR and PLR values by one unit were significantly correlated with LR and DM.

**Table 2:** Univariate and multivariate logistic regression analyses for local recurrence and distant metastasis

	Locoregional recurrence				Distant metastasis			
Parameter	Univariable analysis		Multivariable analysis		Univariable analysis		Multivariable analysis	
	OR (95% CI)	*p*	OR (95% CI)	*p*	OR (95% CI)	*p*	OR (95% CI)	*p*
Age	1.01 (1.01–1.02)	<0.001	1.00 (0.98–1.02)	0.93	1.02 (1.01–1.02)	<0.001	1.00 (0.98–1.03)	0.71
Tumor size	1.10 (1.05–1.15)	<0.001	0.87 (0.75–1.01)	0.07	1.16 (1.10–1.21)	<0.001	0.91 (0.78–1.06)	0.22
FIGO stage	1.89 (1.43–2.48)	<0.001	0.78 (0.33–1.86)	0.57	2.52 (1.88–3.36)	<0.001	0.78 (0.30–2.03)	0.61
Nodal metastasis	3.47 (2.31–5.20)	<0.001	2.06 (1.16–3.67)	0.01	5.70 (3.54–9.17)	<0.001	3.01 (1.61–5.64)	0.001
NLR	1.02 (1.02–1.14)	0.01	1.30 (1.08–1.56)	0.006	1.15 (1.08–1.23)	<0.001	1.32 (1.10–1.54)	0.005
PLR	1.02 (1.01–1.03)	0.004	1.02 (0.98–1.06)	0.37	1.04 (1.02–1.05)	<0.001	1.03 (0.98–1.07)	0.18
Hemoglobin	1.07 (1.05–1.10)	<0.001	1.20 (1.07–1.35)	0.003	1.10 (1.07–1.13)	<0.001	1.16 (1.03–1.32)	0.02

Abbreviations: *OR* odds ratio, *CI* confidence interval, *FIGO* International Federation of Gynecology and Obstetrics, *NLR* neutrophil to lymphocyte ratio, *PLR* platelet to lymphocyte ratio

### Prognostic factors

**Fig. 3:**
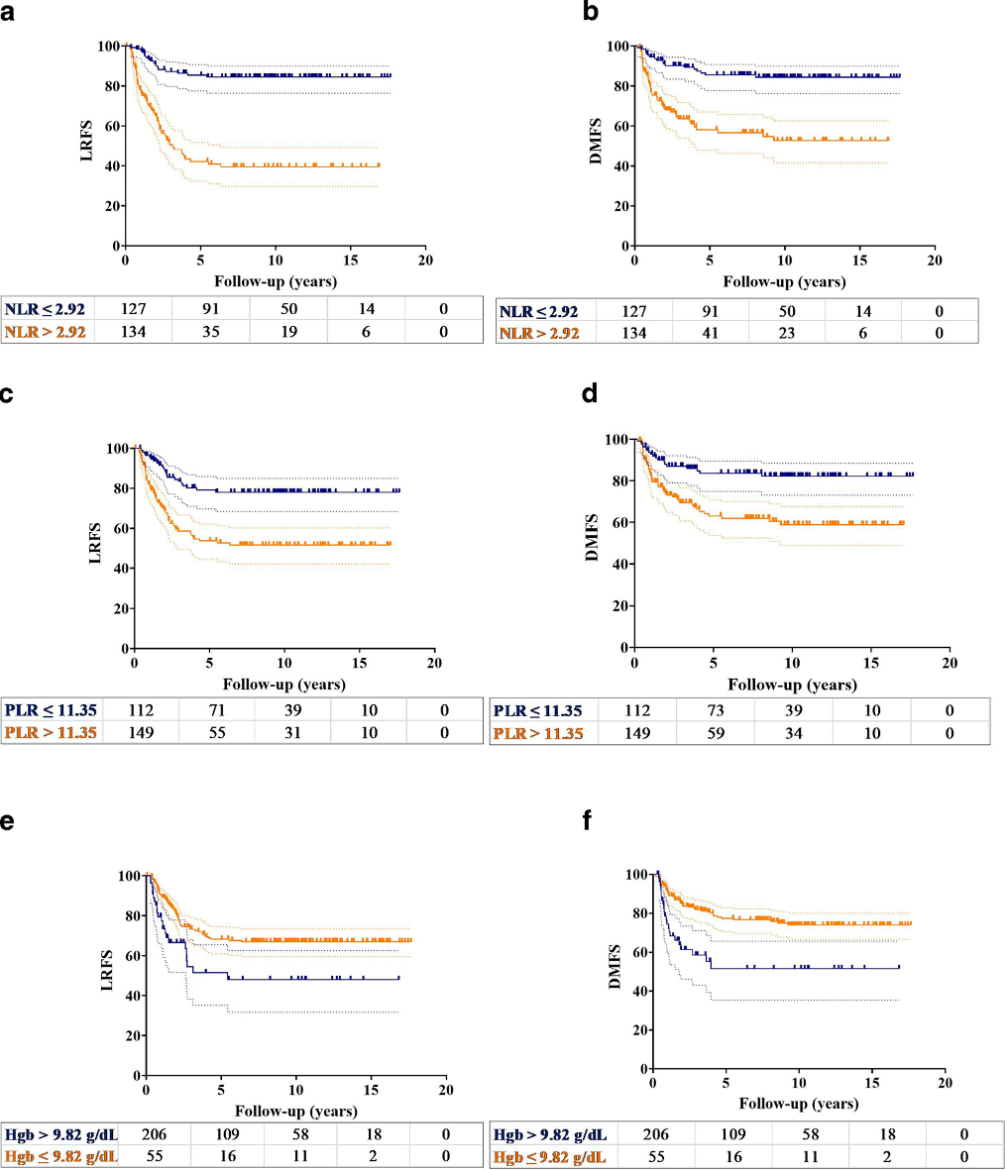
Locoregional recurrence-free survival (LRFS) and (**b**) distant metastasis-free survival (DMFS) curves in patients with NLR ≤2.92 (blue line) and NLR >2.92 (yellow line). (**c**) LRFS and (**d**) DMFS curves in patients with PLR ≤11.35 (blue line) and PLR >11.35 (yellow line). (**e**) LRFS and (**f**) DMFS curves in patients with Hgb >9.82 g/dL (blue line) and Hgb ≤9.82 g/dL (the dashed lines represent 95% confidence interval)

The significant factors for LRFS and DMFS in the univariable analysis were FIGO stage, tumor size, lymph node metastasis, NLR, PLR, and Hgb level (Table 3). The 10-year LRFS rates for patients with NLR ≤2.92 and NLR >2.92 were 84.4 and 39.6% (*p* < 0.001), respectively (Fig. 3a). Similarly, the 10-year LRFS rates were significantly higher in patients with PLR ≤11.35 than in patients with PLR >11.35 (77.9% vs. 51.5%; *p* < 0.001), as well as in patients with Hgb >9.82 g/dL than in those with Hgb ≤9.82 g/dL (67.0% vs. 48.0%; *p* = 0.005) (Fig. 3c, e). The 10-year DMFS were significantly higher in patients with NLR ≤2.92 (84.4% vs. 52.7%; *p* < 0.001), PLR ≤11.35 (82.2% vs. 58.9%; *p* < 0.001), and Hgb >9.83 g/dL (74.0% vs. 51.6%; *p* < 0.001) than in their counterparts (Fig. 3b, d, F).

**Table 3:** Univariable and multivariable analyses of the prognostic factors for locoregional recurrence-free survival (LRFS) and distant metastasis-free survival (DMFS) for the entire cohort

		Locoregional recurrence-free survival				Distant metastasis-free survival			
Variable	Patient no	Univariate analysis		Multivariate analysis		Univariate analysis		Multivariate analysis	
		HR (95% CI)	*p*	HR (95% CI)	*p*	HR (95% CI)	*p*	HR (95% CI)	*p*
*Age*									
< 60 years	153	1	0.35	–	–	1	0.71	–	–
≥ 60 years	108	1.23 (0.80–1.90)	–	–	–	1.10 (0.68–1.77)	–	–	–
*FIGO stage*									
<IIB	36	1	0.01	1	0.08	1	0.03	1	0.21
≥IIB	225	3.13 (1.27–7.75)	–	2.31 (0.91–5.84)	–	3.06 (1.11–8.41)	–	1.93 (0.69–.544)	–
*Tumor size*									
≤ 4 cm	75	1	<0.001	1	0.005	1	<0.001	1	0.11
> 4 cm	186	5.15 (2.49–9.69)	–	2.92 (1.37–6.23)	–	3.80 (1.81–7.95)	–	1.90 (0.87–4.15)	–
*Nodal metastasis*									
Absent	134	1	<0.001	1	0.01	1	<0.001	1	0.001
Present	127	2.76 (1.76–4.33)	–	1.84 (1.16–292)	–	3.55 (2.10–6.01)	–	2.48 (1.44–4.27)	–
*NLR*									
≤ 2.92	127	1	<0.001	1	<0.001	1	<0.001	1	0.002
> 2.92	134	5.39 (3.18–9.12)	–	4.94 (2.51–9.76)	–	3.82 (2.22–6.57)	–	2.94 (1.46–5.91)	–
*PLR*									
≤ 11.35	112	1	<0.001	1	0.29	1	<0.001	1	0.8
> 11.35	149	2.79 (1.71–4.55)	–	1.41 (0.75–2.65)	–	2.61 (1.52–4.48)	–	0.92 (0.46–1.82)	–
*Hemoglobin*									
≤ 9.82 g/dL	55	1	<0.001	1	0.22	1	<0.001	1	0.02
> 9.82 g/dL	206	0.51 (0.31–0.82)	–	1.36 (0.83–2.22)	–	0.40 (0.24–0.67)	–	0.54 (0.32–0.91)	–

Abbreviations: *HR* hazard ratio, *CI* confidence interval, *FIGO* International Federation of Gynecology and Obstetrics, *NLR* neutrophil to lymphocyte ratio, *PLR* platelet to lymphocyte ratio

In the multivariable analysis, large tumor size, presence of lymph node metastasis, and elevated NLR were the independent predictors of poor LRFS and DMFS (Table 3). Serum Hgb level was an additional significant prognostic factor for DMFS.

## Discussion

In this study, where the median follow-up was longer than 10 years, we found that hematological parameters could be used to predict LR and DM in cervical cancer patients undergoing definitive ChRT. The results show a significant correlation of high NLR and low Hgb level with LR and DM. Given that nearly 75% of the patients experienced disease recurrence primarily as DM within two years after ChRT completion, developing effective systemic treatments may be beneficial in improving survival rates. Although NLR and PLR values were significantly higher in patients with LR or DM than in their counterparts, no significant differences in Hgb levels were observed between patients with and without LR or DM. Large tumor size, lymph node metastasis, and elevated NLR were the independent predictors of poor LRFS and DMFS. Serum Hgb level was an additional significant prognostic factor for DMFS.

The concept of hematological parameters has provided a new and profound understanding of the dynamic course of immune-inflammatory responses as a reaction between innate and adaptive cell immune systems in various cancer types, including cervical cancer. These parameters stand out as a simple, inexpensive, and readily available parameter because they reflect the prognosis of cervical cancer patients. NLR, PLR, and Hgb, alone or in combination with other markers, may be useful in the decision-making and management of this patient population. The long-term results of this study, which used hematological parameters as a prognostic marker, provide a wealth of evidence for routine clinical use. Despite the well-documented predictive roles of NLR and anemia in cervical cancer, guidelines do not include these variables.

Chronic inflammation plays an inductive role in carcinogenesis, contributing to the initiation and progression of many cancers [14]. Tumor development, progression, and metastasis can be promoted by an inflammatory microenvironment and inflammatory cells [15, 16]. Neutrophils, lymphocytes, and platelets are immune system components that contribute to tumor progression by releasing reactive oxygen radicals, which can cause DNA damage, genetic instability, and carcinogenesis. An example of an inflammatory process involves prolonged infection with certain types of human papillomavirus (HPV), a condition that leads to cervical cancer. Cancer-associated inflammatory factors trigger neutrophils, and the human immune response to cancer leads to changes in lymphocyte populations, resulting in an elevated NLR [17]. Thus, increased NLR is a systemic response to an inflammatory process, and it appears to be associated with poor prognosis in certain types of cancer [18–20], including cervical cancer [21].

Investigating a cohort of 398 cervical cancer patients who received RT with or without ChT, Cho et al. [22] found that an increased NLR was a significant prognostic indicator for unfavorable LRFS and overall survival (OS). Numerous publications have reported similar unfavorable outcomes in terms of OS and/or progression-free survival (PFS) based on varying thresholds for NLR [11, 17, 23, 24]. Ultimately, the association between high NLR and poor prognoses was reaffirmed by a recent meta-analysis that analyzed data from over ten studies examining the prognostic significance of NLR in cervical cancer patients treated with surgery and/or radiation [9]. Our findings align with those reported in the literature, confirming the potential of NLR as a reliable prognostic indicator in the treatment of cervical cancer. Our study revealed that a high NLR was a significant predictor for LR and DM and for poor LRFS and DMFS.

Platelet count is another indicator of systemic inflammation in a host. Certain proinflammatory cytokines, including IL‑1, IL‑3, and IL‑6, induce thrombopoiesis in cancer patients [25]. The involvement of platelets in mechanisms that promote tumor angiogenesis has been suggested on the basis of their secretion of proangiogenic factors, such as urokinase plasminogen activator and vascular endothelial growth factor [26]. Tumor cells may activate platelets, resulting in the formation of microplatelets that promote tumor invasion [27]. Research has shown that PLR is associated with poor survival and treatment response [10, 11]. By contrast, other studies have found no significant association between PLR and prognosis in cervical cancer patients [17, 28]. Although we found a significant correlation between PLR and LR or DM, we could not establish that PLR had a significant impact on LRFS and DMFS.

The relationship of anemia with recurrence and survival has been a subject of interest, which is spurred by the dogma that anemia contributes to increased tumor hypoxia in humans, as demonstrated in animal models [29]. This association is especially evident in various tumor types and cervical cancer, where anemia is consistently associated with poor local control and low survival rates [30]. Nevertheless, the current body of research on the association between anemia and prognosis in cervical cancer patients offers a considerably variable definition of anemia, as the proposed threshold values for anemia range from 7 to 12 g/dL [12, 30, 31]. In our study, we determined a cut-off value using ROC analysis to differentiate patients with low and high Hgb levels. Our findings suggest that low Hgb levels were predictive for both LR and DM, as well as an independent predictor for poor DMFS. However, no significant association was observed between low Hgb levels and LRFS. There is a clear need for standardized criteria when defining anemia thresholds, as evidenced by the variability in the current definitions of anemia. Our study makes significant contribution by establishing specific Hgb cut-off values that are associated with distinct clinical outcomes in cervical cancer patients.

This study has some limitations. Its retrospective nature is the most notable one. Another is the use of pre-treatment hematological parameters rather than post-treatment parameters to predict recurrence patterns. Future research should also pay attention to the latter. Last, the study focused only on NLR, PLR, and serum Hgb level, not the other inflammatory indices. Despite these limitations, our study has several advantages that render it well suited for assessing the importance of clinical and hematological parameters in determining recurrence patterns; compared with previous studies, our study involved a larger and more homogeneous population, included only those with confirmed SCC histology and those treated with definitive ChRT, and had a significantly longer follow-up period.

## Conclusion

Our study, which spanned over a decade, revealed that hematological parameters have great potential as cost-effective biomarkers for predicting recurrence and survival in cervical cancer patients who undergo definitive ChRT. An independent association was observed between elevated NLR and lower LRFS and DMFS following definitive ChRT, suggesting that the treatment outcomes in cervical cancer patients are influenced by the systemic inflammatory response. Moreover, anemia demonstrated a predictive relationship with LR and DM, thereby highlighting the importance of tumor aggressiveness in individuals exhibiting low Hgb levels. Despite the ease of use and cost-effectiveness of serum hematological parameters as predictive tests for disease recurrence, additional research is warranted to enhance the precision of cervical cancer treatment strategies.
